# Microsatellite instability status determined by next‐generation sequencing and compared with PD‐L1 and tumor mutational burden in 11,348 patients

**DOI:** 10.1002/cam4.1372

**Published:** 2018-02-13

**Authors:** Ari Vanderwalde, David Spetzler, Nianqing Xiao, Zoran Gatalica, John Marshall

**Affiliations:** ^1^ The University of Tennessee Health Science Center and West Cancer Center Memphis Tennessee; ^2^ Caris Life Sciences Phoenix Arizona; ^3^ Lombardi Cancer Center Georgetown University Hospital Washington District of Columbia

**Keywords:** Antineoplastic agents/therapeutic use, checkpoint inhibitor, DNA mismatch repair, microsatellite instability, next‐generation sequencing, precision medicine, programmed cell death 1 receptor/antagonists and inhibitors

## Abstract

Microsatellite instability (MSI) testing identifies patients who may benefit from immune checkpoint inhibitors. We developed an MSI assay that uses data from a commercially available next‐generation sequencing (NGS) panel to determine MSI status. The assay is applicable across cancer types and does not require matched samples from normal tissue. Here, we describe the MSI‐NGS method and explore the relationship of MSI with tumor mutational burden (TMB) and PD‐L1. MSI examined by PCR fragment analysis and NGS was compared for 2189 matched cases. Mismatch repair status by immunohistochemistry was compared to MSI‐NGS for 1986 matched cases. TMB was examined by NGS, and PD‐L1 was determined by immunohistochemistry. Among 2189 matched cases that spanned 26 cancer types, MSI‐NGS, as compared to MSI by PCR fragment analysis, had sensitivity of 95.8% (95% confidence interval [CI] 92.24, 98.08), specificity of 99.4% (95% CI 98.94, 99.69), positive predictive value of 94.5% (95% CI 90.62, 97.14), and negative predictive value of 99.2% (95% CI, 98.75, 99.57). High MSI (MSI‐H) status was identified in 23 of 26 cancer types. Among 11,348 cases examined (including the 2189 matched cases), the overall rates of MSI‐H, TMB‐high, and PD‐L1 positivity were 3.0%, 7.7%, and 25.4%, respectively. Thirty percent of MSI‐H cases were TMB‐low, and only 26% of MSI‐H cases were PD‐L1 positive. The overlap between TMB, MSI, and PD‐L1 differed among cancer types. Only 0.6% of the cases were positive for all three markers. MSI‐H status can be determined by NGS across cancer types. MSI‐H offers distinct data for treatment decisions regarding immune checkpoint inhibitors, in addition to the data available from TMB and PD‐L1.

## Introduction

Microsatellite instability (MSI) involves the gain or loss of nucleotides from microsatellite tracts, which are DNA elements composed of repeating motifs that occur as alleles of variable lengths 1. MSI can result from inherited mutations or originate somatically. Lynch syndrome results from inherited mutations of known mismatch repair (MMR) genes. Tumors are classified as MMR‐deficient (dMMR) if they have somatic or germ line mutations. MSI can also occur due to epigenetic changes or altered microRNA pathways affecting MMR proteins, or without a loss of a known underlying protein 2. MSI is most commonly found in colon and endometrial cancers (the most common Lynch syndrome cancer types); however, recent analyses have found MSI in as many as 24 cancer types, suggesting that MSI is a generalized cancer phenotype 3, 4, 5, 6.

MSI has been associated with improved prognosis, but until the recent advent of immune checkpoint inhibitors, the predictive use of MSI has been limited. A proof‐of‐concept study including 87 patients with 12 different cancer types demonstrated the predictive value of MSI status to predict response of solid tumors to the anti‐PD‐1 agent pembrolizumab 5, 7. This ability of MSI to predict pembrolizumab response has led to the first tumor‐agnostic drug approval by the FDA in May 2017. Additional evidence showed an improved response for MSI‐high (MSI‐H) patients to the anti‐PD‐1 agents nivolumab and MEDI0680, the anti‐PD‐L1 agent durvalumab, and the anti‐CTLA‐4 agent ipilimumab 7, 8, 9, 10. These results elevate MSI status as a third, possibly independent, predictive biomarker for immune checkpoint inhibitors, along with PD‐L1 and tumor mutational burden (TMB) 11, 12, 13, 14, 15, 16, 17. Given that patient responses to these drugs can be highly durable 5, 7, 18, it is critical to identify as many potential responders as possible. Therefore, a method to efficiently determine MSI status for every cancer patient is needed.

Currently, MSI is most commonly detected through polymerase chain reaction (PCR) by fragment analysis (FA) of five conserved satellite regions, which is considered the gold standard method for MSI detection 1, 19. FA is not ideal in the clinic, however, as it requires samples of both tumor and normal tissue. As a result, FA is not always feasible for cases with limited amounts of tissue, including the analysis of cancer metastases, which are commonly submitted as biopsies and may contain few normal cells. Additionally, determining MSI by FA and MMR analysis from immunohistochemistry (IHC) is performed as stand‐alone tests and would be inefficient to perform on every patient with cancer, given that the incidence of MSI is only about 5% across cancer types 5.

As broad tumor profiling becomes a common part of care for patients with cancer, it would be preferable to determine MSI status from sequencing panel results. Next‐generation sequencing (NGS) was recently found to be feasible to determine MSI status, but the published techniques require the use of paired tumor and normal tissue 3, 6. Given a large database of samples with both broad NGS results and matching MSI/dMMR status by FA/IHC, we hypothesized that we could develop and technically validate an NGS‐based MSI assay without the need for matched samples from normal tissue. Here, we describe our process for developing such a method and explore the relationship of MSI with other immunotherapy markers, specifically TMB and PD‐L1.

## Material and Methods

### Patient cohort

For development of the NGS assay, 2189 cases were retrospectively selected based on having data available for both the 592‐gene sequencing panel and MSI testing by PCR‐FA (assay details below). For the TMB, PD‐L1, and MSI‐NGS comparison, 11,348 patients were retrospectively selected based on available data from commercial comprehensive sequencing profiles performed on their tumors by a commercial laboratory (Caris Life Sciences, Phoenix, AZ, USA) that included PD‐L1 by immunohistochemistry (IHC) and the 592‐gene sequencing panel. This research used a collection of existing data that were deidentified prior to analysis. As this research was compliant with 45 CFR 46.101(b), the project was deemed exempt from IRB oversight and consent requirements were waived.

### Fragment analysis by PCR

MSI‐FA was tested by the fluorescent multiplex PCR‐based method (MSI Analysis: Promega, Life Sciences, Madison, WI, USA).

### Next‐generation sequencing

NGS was performed on genomic DNA isolated from formalin‐fixed paraffin‐embedded (FFPE) tumor samples using the NextSeq platform (Illumina Inc., San Diego, CA, USA). A custom‐designed SureSelect XT assay (Agilent Technologies, Santa Clara, CA, USA) was used to enrich the 592 whole‐gene targets that comprised a 592‐gene NGS panel. All variants were detected with >99% confidence based on allele frequency and baited capture pull‐down coverage with an average sequencing depth of over 500X and an analytic sensitivity of 5% variant frequency.

### Microsatellite instability by NGS

Microsatellite loci in the target regions of a 592‐gene NGS panel were first identified using the MISA algorithm (pgrc.ipk‐gatersleben.de/misa/), which revealed 8921 microsatellite locations. Subsequent analyses excluded sex chromosome loci, microsatellite loci in regions that typically have lower coverage depth relative to other genomic regions, and microsatellites with repeat unit lengths greater than five nucleotides. These exclusions resulted in 7317 target microsatellite loci.

Patient DNA was sequenced by NGS using the 592‐gene panel. We examined the 7317 target microsatellite loci and compared them to the reference genome hg19 from the UCSC Genome Browser database (http://hgdownload.cse.ucsc.edu/goldenPath/hg19/bigZips/). The number of microsatellite loci that were altered by somatic insertion or deletion was counted for each patient sample. Only insertions or deletions that increased or decreased the number of repeats were considered. A locus was not counted more than once even if it had multiple lengths of insertions or deletions. Thresholds were calibrated based on comparison of total number of altered loci per patient to MSI‐FA results with the aim to maximize sensitivity while maintaining an appropriately high specificity, positive predictive value (PPV), and negative predictive value (NPV).

### Total mutation burden

TMB was calculated based on the number of nonsynonymous somatic mutations identified by NGS while excluding any known single nucleotide polymorphisms (SNPs) in dbSNP (version 137) or in the 1000 Genomes Project database (phase 3; http://www.internationalgenome.org/) 20. TMB is reported as mutations per Mb sequenced. The threshold for determining high TMB as greater than or equal to 17 mutations/megabase was established by comparing TMB with MSI by FA in CRC cases, based on reports of TMB having high concordance with MSI in CRC 7, 21.

### PD‐L1 IHC

IHC analysis was performed on slides of FFPE tumor samples using automated staining techniques. The procedures met the standards and requirements of the College of American Pathologists.

The primary antibody against PD‐L1 was SP142 (Spring Bioscience, Pleasanton, CA, USA), except for NSCLC tumors tested after January 2016. For NSCLC tumors tested after January 2016, the primary PD‐L1 antibody clone was 22c3 (Dako, Santa Clara, CA, USA). For the calculations in this manuscript, staining for both antibodies was considered positive if there was staining on ≥1% of tumor cells.

### Mismatch repair protein IHC

MMR protein expression was tested by IHC using antibody clones (MLH1, M1 antibody; MSH2, G2191129 antibody; MSH6, 44 antibody; PMS2, EPR3947 antibody [Ventana Medical Systems, Inc., Tucson, AZ, USA]). The complete absence of protein expression (0+ in 100% of cells) was considered a loss of MMR and thus dMMR.

### Cancer types analyzed by PCR‐FA and by MSI‐NGS

Matched cases analyzed both by PCR‐FA and by MSI‐NGS included the following cancer types: bladder cancer (*n* = 3), breast carcinoma (*n* = 16), cervical cancer (*n* = 2), cholangiocarcinoma (*n* = 17), colorectal adenocarcinoma (*n* = 1193), endometrial cancer (*n* = 708), esophageal and esophagogastric junction carcinoma (*n* = 7), extrahepatic bile duct adenocarcinoma (*n* = 2), gastric adenocarcinoma (*n* = 10), gastrointestinal stromal tumors (*n* = 2), glioblastoma (*n* = 9), liver hepatocellular carcinoma (*n* = 8), lymphoma (*n* = 2), malignant solitary fibrous tumor of the pleura (*n* = 1), melanoma (*n* = 4), neuroendocrine tumors (*n* = 10), none of these apply (*n* = 21), NSCLC (*n* = 5), other female genital tract malignancy (*n* = 12), ovarian surface epithelial carcinomas (*n* = 15), pancreatic adenocarcinoma (*n* = 44), prostatic adenocarcinoma (*n* = 1), small intestinal malignancies (*n* = 7), soft tissue tumors (*n* = 1), thyroid carcinoma (*n* = 1), uterine sarcoma (*n* = 87), and uveal melanoma (*n* = 1).

### Cancer types analyzed by IHC and by MSI‐NGS

Matched cases analyzed both by IHC and by MSI‐NGS included the following cancer types: bladder cancer (*n* = 4), breast carcinoma (*n* = 18), cervical cancer (*n* = 1), cholangiocarcinoma (*n* = 21), colorectal adenocarcinoma (*n* = 925), endometrial cancer (*n* = 445), esophageal and esophagogastric junction carcinoma (*n* = 8), gastric adenocarcinoma (*n* = 15), gastrointestinal stromal tumors (*n* = 3), glioblastoma (*n* = 53), head and neck squamous cell carcinoma (*n* = 1), kidney cancer (*n* = 1), liver hepatocellular carcinoma (*n* = 12), low‐grade glioma (*n* = 7), lymphoma (*n* = 3), melanoma (*n* = 2), neuroendocrine tumors (*n* = 10), none of these apply (*n* = 38), NSCLC (*n* = 6), other female genital tract malignancy (*n* = 3), ovarian surface epithelial carcinomas (*n* = 17), pancreatic adenocarcinoma (*n* = 318), prostatic adenocarcinoma (*n* = 2), small intestinal malignancies (*n* = 5), soft tissue tumors (*n* = 1), uterine sarcoma (*n* = 65), and uveal melanoma (*n* = 2).

## Results

**Figure 1:**
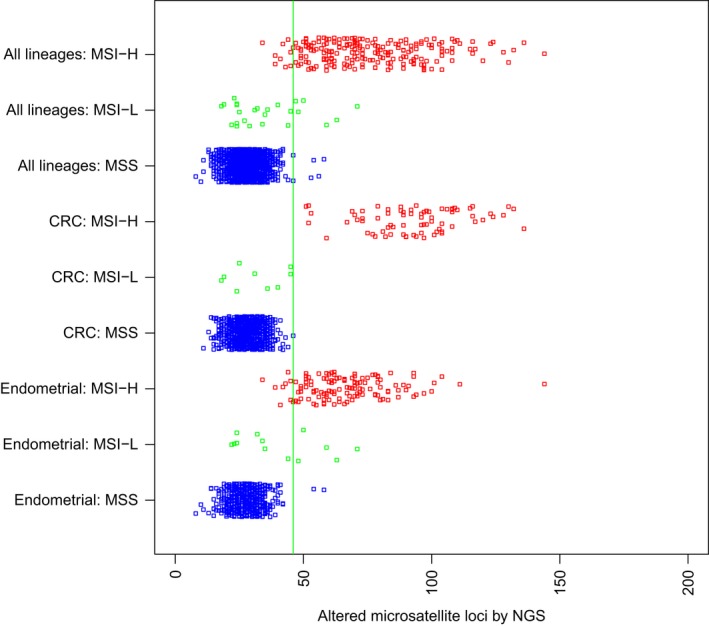
Analysis by PCR‐FA (*y*‐axis) classified cases as MSS (blue), MSI‐low (green), or MSI‐H (red), and NGS (*x*‐axis) classified cases as MSS (<46 altered microsatellite loci/Mb) or MSI‐H (≥46 altered microsatellite loci/Mb). Cases include 26 different cancer types (*n* = 2189), colorectal adenocarcinoma (*n* = 1193), and endometrial cancer (*n* = 708). Abbreviations: Mb, megabase; MSI‐H, microsatellite high; MSI‐L, microsatellite low; MSS, microsatellite stable; NGS, next‐generation sequencing.

Matched MSI‐FA PCR and 592‐gene NGS assays from 2189 cases (Fig. 1 and Table 1) were used to calibrate the MSI‐NGS assay to classify samples as MSI‐H or microsatellite stable (MSS). A cutoff of ≥46 altered loci was chosen with the goal of optimizing the performance of the MSI‐NGS test in CRC and endometrial cancers, which are the cancer types for which MSI testing has traditionally had the highest clinical relevance (Fig. 1). Lower cutoffs resulted in unacceptably high levels of MSS‐FA CRC cases (Fig. 1). Good performance was maintained when this cutoff was used across all 2189 FA‐matched cases that spanned 26 cancer types (sensitivity 95.8% [95% confidence interval (CI) 92.24, 98.08], specificity 99.4% [95% CI 98.94, 99.69], PPV 94.5% [95% CI 90.62, 97.14], and NPV 99.2% [95% CI, 98.75, 99.57]). For the purposes of calculating the MSI‐NGS performance metrics, cases categorized as MSI‐low (MSI‐L) by FA were grouped with the MSS‐FA cohort. As patients with MSI‐L tumors are most often treated in a manner similar to patients with MSS tumors in the clinic, grouping MSI‐L with MSS is reasonable.

**Table 1:** Classification of MSI by next‐generation sequencing compared with PCR fragment analysis for 2189 matched cases

			Next‐generation sequencing					
			MSI‐H	MSS	Sensitivity % (95% CI)	Specificity % (95% CI)	PPV % (95% CI)	NPV % (95% CI)
			No. of patients					
All types of cancer (FA data for *n* = 2189)	Fragment analysis	MSS	6	1941	95.8 (92.24, 98.08)	99.4 (98.94, 99.69)	94.5 (90.62, 97.14)	99.2 (98.75, 99.57)
		MSI‐L	6	20				
		MSI‐H	207	9				
Colorectal cancer (FA data for *n* = 1193)		MSS	1	1108	100.0 (95.2, 100)	99.9 (99.5, 100)	98.7 (92.89, 99.97)	99.6 (99.09, 99.9)
		MSI‐L	0	9				
		MSI‐H	75	0				
Noncolorectal cancer (FA data for *n* = 996)		MSS	5	833	93.6 (88.23, 97.04)	98.7 (97.71, 99.36)	92.3 (86.65, 96.1)	98.7 (97.71, 99.36)
		MSI‐L	6	11				
		MSI‐H	132	9				
Endometrial cancer (FA data for *n* = 709)		MSS	2	562	93.9 (88.32, 97.33)	98.8 (97.52, 99.51)	94.6 (89.22, 97.81)	98.4 (97.07, 99.29)
		MSI‐L	5	8				
		MSI‐H	123	8				

CRC, colorectal cancer; FA, fragment analysis; MMR, mismatch repair; MSI‐L, microsatellite instability‐low; MSI‐H, microsatellite instability‐high; MSS, microsatellite stable; NGS, next‐generation sequencing; NPV, negative predictive value; PPV, positive predictive value.

An additional comparison involved 1986 cases that were examined both by MSI‐NGS and by IHC for MMR protein status (Table 2). Cases with dMMR protein status were identified by IHC in 171 cases (8.6%), while NGS identified 156 cases (7.9%). Compared with IHC for MMR proteins, across 26 cancer types, NGS had a sensitivity of 87.1%, specificity of 99.6%, PPV of 95.5%, and NPV of 98.8%. Compared with IHC for MMR proteins, NGS of CRC cases had a sensitivity of 91.7%, specificity of 99.7%, PPV of 94.8%, and NPV of 99.4%.

**Table 2:** Classification of microsatellite instability by next‐generation sequencing compared with MMR by IHC

			Next‐generation sequencing					
			MSI‐H	MSS	Sensitivity (%)	Specificity (%)	PPV (%)	NPV (%)
			No. of patients					
All types of cancer (*n* = 1986)	IHC MMR	dMMR	149	22	87.1	99.6	95.5	98.8
		MMR‐P	7	1808				
CRC (*n* = 925)		dMMR	55	5	91.7	99.7	94.8	99.4
		MMR‐P	3	862				
Non‐CRC (*n* = 1061)		dMMR	94	17	84.7	99.6	95.9	98.2
		MMR‐P	4	946				

IHC, immunohistochemistry; MMR, mismatch repair; dMMR, deficient mismatch repair; MMR‐P, mismatch repair proficient; MSI‐H, microsatellite instability‐high; MSS, microsatellite stable; NPV, negative predictive value; PPV, positive predictive value.

The highest percentage of MSI‐H cases was endometrial cancer (17%; Table 3), followed by gastric adenocarcinoma (9%), small intestinal malignancies (8%), and colorectal adenocarcinoma (6%). Cancer types with no cases of MSI‐H included melanoma (0 of 360 cases), bladder cancer (0 of 144), head and neck squamous carcinoma (0 of 118), low‐grade glioma (0 of 107), gastrointestinal stromal cancers (0 of 65), and thymic cancer (0 of 28).

**Table 3:** Biomarkers by NGS across cancer types

	*N*	MSI		TMB		PD‐L1		MSI + TMB		MSI + PD‐L1		TMB + PD‐L1		MSI + TMB + PD‐L1		None of these biomarkers	
		*n*	%	*n*	%	*n*	%	*n*	%	*n*	%	*n*	%	*n*	%	*n*	%
All cancer types	11348	342	3.0	877	7.7	2887	25.4	240	2.1	89	0.8	390	3.4	71	0.6	7890	69.5
NSCLC	1868	12	0.6	264	14.1	1013	54.2	9	0.5	8	0.4	143	7.7	6	0.3	733	39.2
Ovarian surface epithelial carcinomas	1517	17	1.1	24	1.6	291	19.2	13	0.9	6	0.4	10	0.7	6	0.4	1208	79.6
Colorectal adenocarcinoma	1395	80	5.7	93	6.7	100	7.2	76	5.4	23	1.6	24	1.7	22	1.6	1223	87.7
Breast carcinoma	1024	6	0.6	31	3.0	99	9.7	4	0.4	1	0.1	4	0.4	1	0.1	896	87.5
Endometrial carcinoma	879	155	17.6	110	12.5	142	16.2	89	10.1	24	2.7	22	2.5	15	1.7	592	67.3
None of these apply	705	7	1.0	91	12.9	219	31.1	4	0.6	2	0.3	54	7.7	1	0.1	447	63.4
Pancreatic adenocarcinoma	518	6	1.2	6	1.2	112	21.6	4	0.8	3	0.6	2	0.4	2	0.4	401	77.4
Glioblastoma	427	3	0.7	15	3.5	106	24.8	3	0.7	1	0.2	5	1.2	1	0.2	311	72.8
Melanoma	345	0	0.0	126	36.5	146	42.3	0	0.0	0	0.0	66	19.1	0	0.0	139	40.3
Soft tissue tumors	283	1	0.4	11	3.9	59	20.8	0	0.0	0	0.0	7	2.5	0	0.0	219	77.4
Neuroendocrine tumors	193	7	3.6	7	3.6	16	8.3	3	1.6	2	1.0	3	1.6	2	1.0	169	87.6
Prostatic adenocarcinoma	191	4	2.1	5	2.6	13	6.8	4	2.1	1	0.5	1	0.5	1	0.5	174	91.1
Esophageal and esophagogastric junction carcinoma	189	0	0.0	1	0.5	47	24.9	0	0.0	0	0.0	1	0.5	0	0.0	142	75.1
Gastric adenocarcinoma	184	16	8.7	16	8.7	34	18.5	15	8.2	8	4.3	9	4.9	8	4.3	142	77.2
Cholangiocarcinoma	177	4	2.3	6	3.4	33	18.6	3	1.7	1	0.6	1	0.6	0	0.0	139	78.5
Cervical cancer	168	6	3.6	13	7.7	74	44.0	2	1.2	3	1.8	10	6.0	1	0.6	89	53.0
Kidney cancer	155	1	0.6	1	0.6	46	29.7	0	0.0	1	0.6	0	0.0	0	0.0	108	69.7
Bladder cancer	143	0	0.0	24	16.8	61	42.7	0	0.0	0	0.0	9	6.3	0	0.0	67	46.9
Uterine sarcoma	128	3	2.3	3	2.3	24	18.8	1	0.8	1	0.8	3	2.3	1	0.8	102	79.7
Head and neck squamous carcinoma	111	0	0.0	6	5.4	72	64.9	0	0.0	0	0.0	4	3.6	0	0.0	37	33.3
Low‐grade glioma	95	0	0.0	1	1.1	7	7.4	0	0.0	0	0.0	0	0.0	0	0.0	87	91.6
Small cell lung cancer	75	1	1.3	4	5.3	10	13.3	0	0.0	0	0.0	2	2.7	0	0.0	62	82.7
Liver hepatocellular carcinoma	73	2	2.7	1	1.4	7	9.6	1	1.4	0	0.0	0	0.0	0	0.0	64	87.7
Small intestinal malignancies	72	6	8.3	6	8.3	12	16.7	5	6.9	1	1.4	1	1.4	1	1.4	54	75.0
Other female genital tract malignancies	57	1	1.8	4	7.0	27	47.4	1	1.8	0	0.0	3	5.3	0	0.0	29	50.9
Nonepithelial ovarian cancer	56	1	1.8	0	0.0	4	7.1	0	0.0	0	0.0	0	0.0	0	0.0	51	91.1
Gastrointestinal stromal tumors	52	0	0.0	0	0.0	20	38.5	0	0.0	0	0.0	0	0.0	0	0.0	32	61.5
Uveal melanoma	50	1	2.0	1	2.0	10	20.0	1	2.0	1	2.0	1	2.0	1	2.0	40	80.0
Retroperitoneal or peritoneal sarcoma	46	0	0.0	0	0.0	10	21.7	0	0.0	0	0.0	0	0.0	0	0.0	36	78.3
Thyroid carcinoma	42	1	2.4	1	2.4	26	61.9	1	2.4	1	2.4	1	2.4	1	2.4	16	38.1
Extrahepatic bile duct adenocarcinoma	29	1	3.4	1	3.4	6	20.7	1	3.4	1	3.4	1	3.4	1	3.4	23	79.3
Lymphoma	27	0	0.0	2	7.4	16	59.3	0	0.0	0	0.0	2	7.4	0	0.0	11	40.7
Thymic carcinoma	26	0	0.0	1	3.8	18	69.2	0	0.0	0	0.0	1	3.8	0	0.0	8	30.8
Male genital tract malignancy	15	0	0.0	0	0.0	3	20.0	0	0.0	0	0.0	0	0.0	0	0.0	12	80.0
Multiple myeloma	10	0	0.0	0	0.0	0	0.0	0	0.0	0	0.0	0	0.0	0	0.0	10	100.0
Retroperitoneal or peritoneal carcinoma	7	0	0.0	0	0.0	2	28.6	0	0.0	0	0.0	0	0.0	0	0.0	5	71.4
Merkel cell carcinoma	6	0	0.0	2	33.3	0	0.0	0	0.0	0	0.0	0	0.0	0	0.0	4	66.7
Nodal diffuse large B‐cell lymphoma	5	0	0.0	0	0.0	2	40.0	0	0.0	0	0.0	0	0.0	0	0.0	3	60.0
Malignant solitary fibrous tumor of the pleura	3	0	0.0	0	0.0	0	0.0	0	0.0	0	0.0	0	0.0	0	0.0	3	100.0
Acute myeloid leukemia	1	0	0.0	0	0.0	0	0.0	0	0.0	0	0.0	0	0.0	0	0.0	1	100.0
Lung bronchioloalveolar carcinoma	1	0	0.0	0	0.0	0	0.0	0	0.0	0	0.0	0	0.0	0	0.0	1	100.0

**Figure 2:**
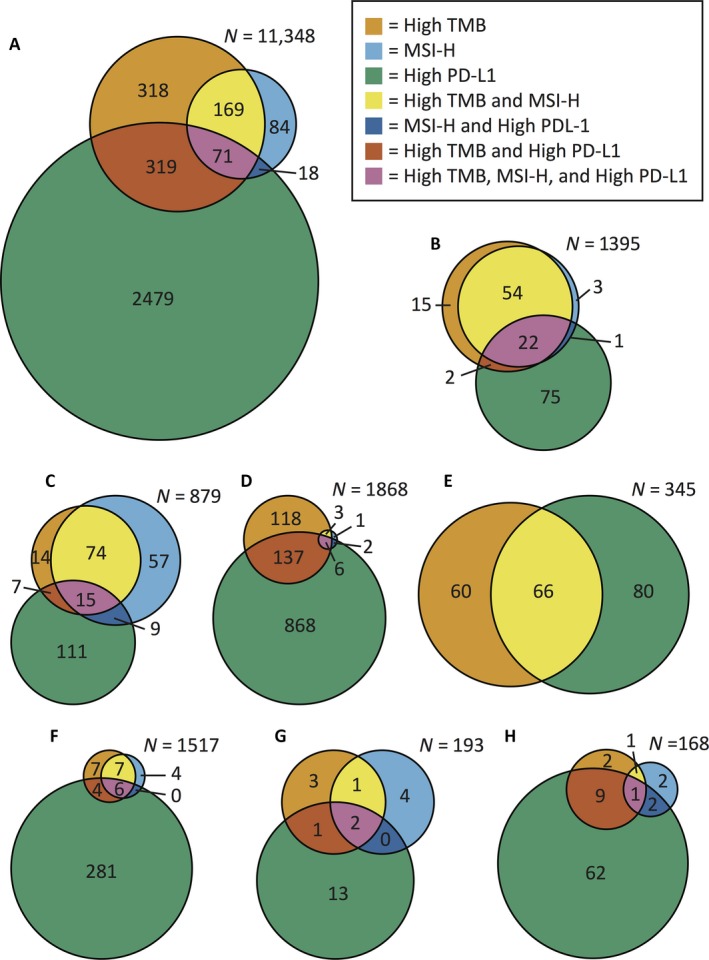
Venn diagrams of the relationships between high TMB, MSI‐H, and high PD‐L1 for (A) all cancer types, (B) CRC, (C) endometrial cancer, (D) NSCLC, (E) melanoma, (F) ovarian surface epithelial carcinomas, (G) neuroendocrine tumors, and (H) cervical cancer. Each *n* value indicates the total number of cases of that cancer type. Abbreviations: MSI‐H, microsatellite high; TMB, tumor mutational burden; PD‐L1, programmed death ligand 1.

The relationship between TMB, MSI, and PD‐L1 was explored by analyzing 11,348 cases that had results for all three assays (Fig. 2A and Table 3). In this set, the overall rate of MSI‐H was 3.0%. Overall, high TMB was 7.7%, and PD‐L1 positivity was 25.4%. Among MSI‐H cases, 70% were also high TMB (62.6% with CRC cases removed). Among high TMB cases, 27% were also MSI‐H. Only 0.6% of the cases were positive for all three markers, while 69.5% of the cases were negative for all three. Of the total cohort, only 26% of MSI‐H cases were PD‐L1 positive compared to 44% of high TMB cases.

The overlap between the biomarkers TMB, MSI, and PD‐L1 differed among cancer types (Fig. 2B–H). High TMB and MSI‐H had 95% overlap for CRC, which was expected, as the TMB cutoff was based on CRC MSI‐FA results. However, only 57% of MSI‐H endometrial cancer cases were also high TMB. Likewise, ovarian, neuroendocrine, and cervical cancers also had significant percentages of MSI‐H cases that were not TMB‐high. In contrast, NSCLC and melanoma had few or no MSI‐H cases, while still having a significant number of high TMB cases.

**Figure 3:**
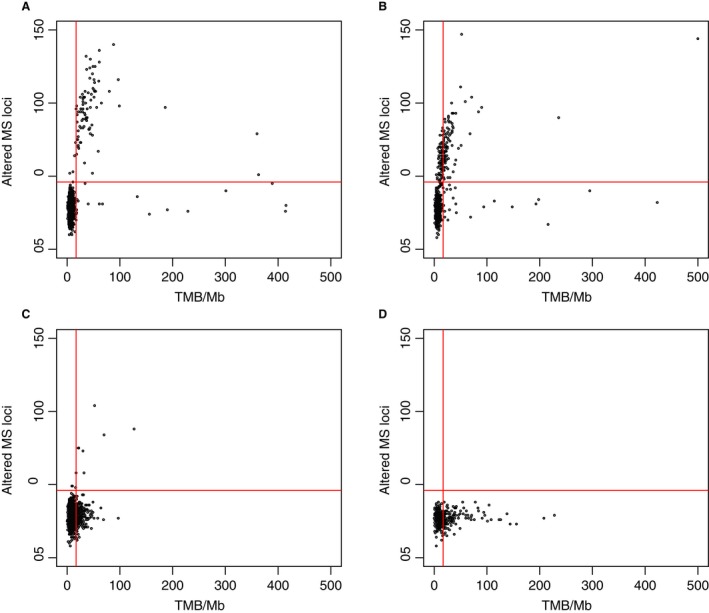
Scatter plots comparing MSI as altered microsatellite (MS) loci determined by NGS to TMB per megabase for (A) colorectal adenocarcinoma (*n* = 1267), (B) endometrial cancer (*n* = 667), (C) NSCLC (*n* = 964), and (D) melanoma (*n* = 175). The horizontal line indicates 46 altered MS, and the vertical line indicates 17 mutations/Mb.

Certain cancer types showed interesting relationships regarding MSI and TMB (Fig. 3). In both CRC and endometrial cancer, the majority of MSI‐H cases were also high in TMB. This pattern was not seen in two cancer types driven primarily by environmentally caused mutagenesis. In NSCLC, 14.1% (264/1868) of cases were high TMB, but only 0.6% (12/1868) were MSI‐H. Notably, melanoma had no cases that were MSI‐H, but had many cases with high TMB (36.5% [126/345]).

## Discussion

MSI‐H cancers are a genetically defined subset of cancers with the potential for enhanced responsiveness to anti‐PD‐1 therapies 5, 6, 7. Determining MSI status across cancer types offers the opportunity to identify patients who are likely to respond while avoiding unnecessary toxicities for patients identified as unlikely to respond. Here, we show the development of a sensitive and specific MSI assay by NGS that is comparable to the existing gold standard of PCR‐FA methods without requiring matched samples from normal tissue.

The method was calibrated with 2189 cases across 26 cancer types that had both MSI‐FA and 592‐gene NGS results. This number of matched samples between FA and NGS is a substantially larger calibration set than that used in another published NGS‐MSI method 22. Previously published data using the MSI‐NGS method described herein found MSI‐H status present in 24 of 31 cancer types 23. Likewise, here, we identified MSI‐H in 23 of 26 cancer types. The detection of MSI‐H cases in this extensive list of cancer types supports the concept that MSI may be a generalized cancer phenotype 3.

Notably, MSI‐H cases that were not TMB‐H or PD‐L1‐positive occurred in significant percentages of ovarian (24%), neuroendocrine (57%), and cervical (33%) cancers. With the recent approval of pembrolizumab for MSI‐H patients of any solid tumor type, this subset of patients now has a promising treatment that would not have been identified using either of the other two immunotherapy biomarker assays. Given the lack of overlap of MSI and high TMB in several cancer types, these data do not support substituting TMB analysis with MSI‐NGS or vice versa. If future clinical studies show significantly reduced response rates of TMB‐low/MSI‐H or TMB‐high/MSS tumors to pembrolizumab, then this conclusion can be reconsidered.

This MSI‐NGS assay has good concordance with the FA method for CRC (100% sensitivity and 99.9% specificity), but its performance is slightly reduced when looking across all cancer types (95.8% sensitivity and 99.9% specificity; PPV of 94.5%). As the FA test was developed for CRC, MSI‐NGS discrepancies in non‐CRC cancer types may be due to other loci being involved in these cancer types that are not measured by the FA method. This raises the possibility that some of the FA PCR results could be false negatives, rather than the corresponding MSI‐NGS results being false positives. Future studies investigating responses to immunotherapies in these discordant patients will help to identify the clinical relevance of these discrepancies. This NGS assay, with broader microsatellite coverage, may be a better predictor of response than the FA assay, which is limited to five microsatellite sites.

The use of NGS to determine MSI status offers significant advantages over FA by PCR. Due to the large number of microsatellite regions analyzed, this method of NGS analysis of MSI does not require a sample of normal tissue for comparison. The comparison of a large number of microsatellite sequences to a reference human genome was able to provide a level of sensitivity comparable to that achieved using only a few microsatellites and comparing to a normal sample from the same patient. Thus, with this method, it is feasible to determine MSI status for patients who do not have available normal tissue or for whom it would be a burden to obtain. Coupling the calculation of MSI to data that are already generated by a broad NGS panel allows for MSI status to be determined efficiently for any patient who is already receiving broad NGS results, without adding the cost of an additional stand‐alone test or consuming additional tumor tissue that could be used for other testings. Further, while FA by PCR was optimized to analyze CRC 24, our NGS analysis of MSI is a pan‐cancer method whose development was technically validated across 26 cancer types.

IHC testing for MMR protein is commonly performed on CRC and endometrial cancer cases to test for Lynch syndrome. Clinical evidence indicates that treatments with the PD‐1 inhibitors pembrolizumab and nivolumab both lead to favorable responses in patients with dMMR tumors 5, 7, 18. Our NGS‐MSI assay has only 87.1% sensitivity for dMMR detection compared to MMR‐IHC (Table 2). However, the proteins measured by standard MMR‐IHC (MLH1, MSH2, MSH6, and PMS2) are not equal in their contribution to the mismatch repair process. Previous research on endometrial carcinoma found that most MSI‐H tumors had loss of MLH1 and PMS2, with concordant loss of the MLH1/PMS2 heterodimer in 48% and with MSI‐H in 97% of PSM2‐negative cases 25. As such, there may be a subset of dMMR cases with relatively low microsatellite alterations, which are identified as MSS by NGS, that have lower rates of response to PD‐1 inhibition compared with cases that are MSI‐H and dMMR cases. This hypothesis is supported by data indicating that the subset of dMMR CRC cases called MSS by FA was much less likely to respond to nivolumab than MSI‐H cases 18. Until more data are available, the best choice may be to run both MSI‐NGS and MMR‐IHC, in lineages where MMR‐IHC loss is more common, to identify as many patients as possible. In addition to this question of magnitude of clinical response for dMMR/MSS patients, MMR‐IHC testing may be impractical for malignancies with low rates of microsatellite instability as these tests require dedicated slides, consuming valuable tissue for a low yield of pathogenic findings.

Current NCCN guidelines recommend MSI and MMR proficiency testing on patients with colon and endometrial cancer. Considering the landscape of the site‐agnostic approval of pembrolizumab for patients with MSI‐H cancers, the testing recommendation should now be expanded to include all patients with advanced solid tumors lacking satisfactory treatment options. The method of MSI‐NGS addresses many of the disadvantages of both FA and MMR‐IHC, thus providing an ideal platform to measure MSI status in all tumors. MSI‐NGS is easily added to other malignancy‐specific molecular panels, requires no extra tissue, and has lower marginal cost when FA is considered as an add‐on test that must be performed along with an NGS panel. With the evolution in cancer care toward molecularly defined diagnoses, validation of NGS measurement of MSI status provides a needed mechanism for all patients with cancer, regardless of malignancy, to achieve testing that can determine whether a potentially life‐extending agent may be appropriate.

A question remains regarding the clinical relevance of measuring both TMB and MSI. MSI is measured by NGS through counting insertions or deletions of 2–5 nucleotides in specific areas of the genome known to accumulate errors in microsatellites. In contrast, TMB was measured here by counting nonsynonymous mutations across the sequenced portion of the genome. Therefore, TMB can capture a wider range of mutational signatures because it covers the genome more broadly. While most MSI‐H cases are high TMB, the opposite is not true. The comparison here of TMB and MSI in CRC is limited by the fact that the threshold for TMB was based on the CRC MSI‐FA results. Our cutoff for high TMB of ≥17 mutations/Mb is similar to the recently published cutoff values of >13.8 and >20 mutations/Mb 6, 26. True biological differences in TMB and MSI appear to exist in certain cancer types. For example, tumors driven primarily by environmentally caused mutations (NSCLC and melanoma) have a much higher proportion of cases with high TMB than MSI (Fig. 3) compared to tumors that are not as strongly associated with environmental causes.

Potential selection bias may limit the ability to extrapolate from this study. The 11,348 cases included in these comprehensive genomic analyses by NGS are generally from patients with advanced, refractory disease who lacked obvious treatment options. This could lead to some bias in the reported MSI frequencies, for example, CRC MSI‐H rates are lower in advanced disease than in the overall CRC population 4.

In conclusion, we have used a large database to develop a method to determine MSI status using NGS results. Specifically, this MSI‐NGS method is applicable across cancer types and does not require matched normal samples, thus providing an alternative for patients with limited tissue. The investigation of the relationship among TMB, MSI, and PD‐L1 revealed a population with MSI‐H disease, but low TMB and no PD‐L1 expression, thus expanding the pool of potential immunotherapy recipients. Until more clinical data are available to show how these markers work together, the best option may be to continue to measure all three to ensure that as many patients as possible benefit from these drugs.
