# Corrigendum

**DOI:** 10.1002/cam4.1593

**Published:** 2018-06-20

Microsatellite instability status determined by next‐generation sequencing and compared with PD‐L1 and tumor mutational burden in 11 348 patients

Ari Vanderwalde, David Spetzler, Nianqing Xiao, Zoran Gatalica & John Marshall

Cancer Medicine 2018; 7: 746‐756

The published version of Figure 2E coloration of this article was incorrect. The correct Figure 2 is shown below:

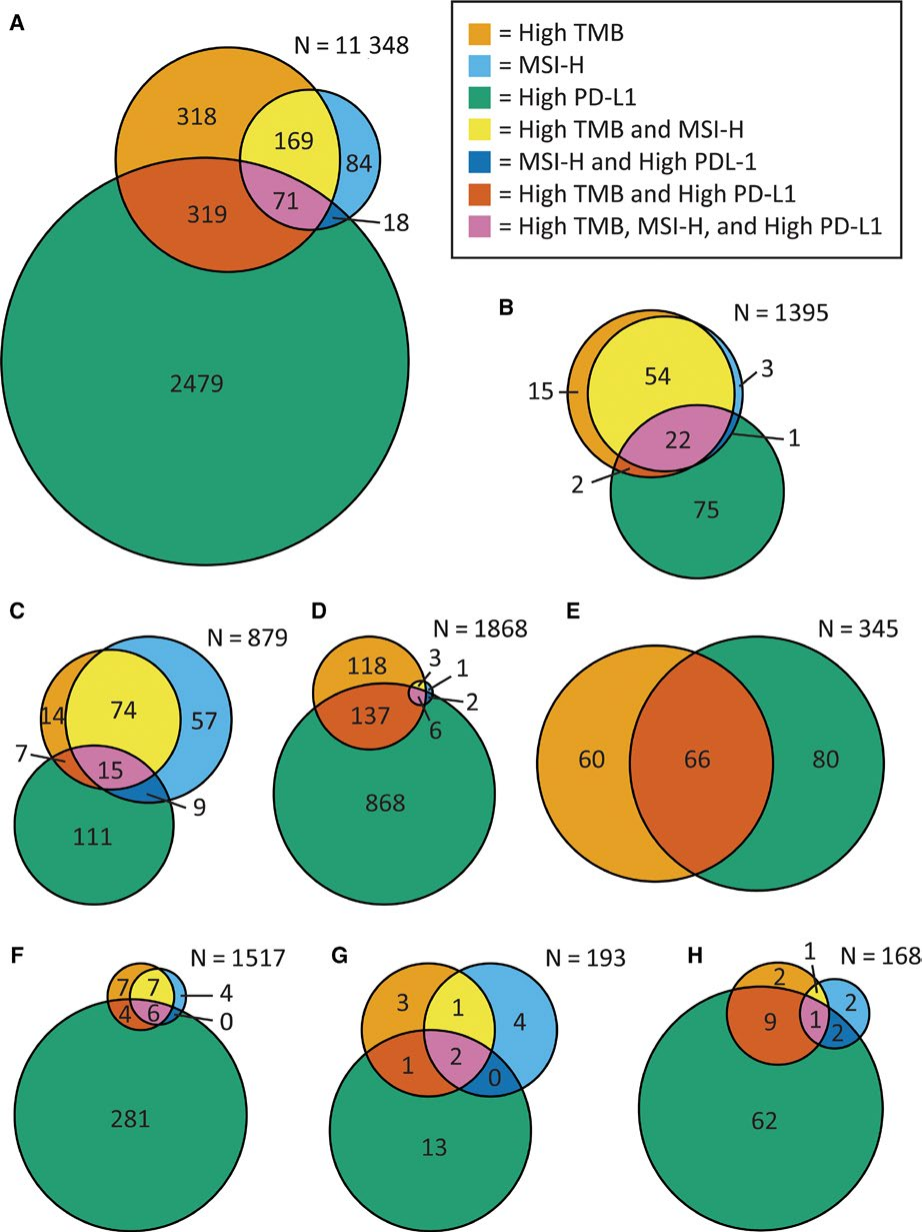

The corresponding author apologizes for this error.

Vanderwalde A, Spetzler D, Xiao N, Gatalica Z, Marshall J. Microsatellite instability status determined by next‐generation sequencing and compared with PD‐L1 and tumor mutational burden in 11 348 patients. *Cancer Med*. 2018;7:746‐756.

